# A Discourse on the Times, Character, and Writings of Hippocrates

**Published:** 1853-01

**Authors:** 


A Discourse on the Times, Character, and Writings of Hippocrates, read before the Trustees, Faculty, and Medical Class at the College of Physicians and Surgeons, at the opening of the term of 1852—53, by Elisha Bartlett. M. D., Professor of Materia Medica and Medical Jurisprudence.

THE opening of the sessions of the various Medical Colleges furnishes occasion for display of the literary talent of the several professors. Many of the introductories which we have received are creditable alike to their authors and the schools which they represent. If, however, we may be allowed to express our opinion, we should say that too much prominence is given to the evils which have crept into the profession, and too much time spent in deploring the disabilities under which we labor. The result is, that the non-medical, and, to a certain extent, the medical public have come to the conclusion that our art is deteriorated, and that our science is a tissue of speculations. To correct this, we want faithful pictures from early history, by which we may know the relations which the physician has sustained to, and the estimation in which he has been held by the masses of the people.

In the discourse of Professor Bartlett, we have introduced to our acquaintance the Father of Historic Medicine. We are sure that our readers will be gratified with the picture of the author; we therefore make no apology for the length of the extract.

"In one of the years of the 88th Olympiad, in the island of Thasos, fronting the Thracian city of Abdera, there was sadness in the house of Silenus, for its young master had been seized with sudden and alarming illness:—the fiery *causus* of the climate. . . . . . . . . . . . It is the third day of his disease; he has had a restless and distressed night, with some wandering of the mind; the symptoms are all worse in the morning, and his family and neighbors are anxious and alarmed. The occupations and order of the old Thracian household are interrupted and broken up. A fresh offering has been placed on the altar of the household Jove, standing in the centre of the inner court. The sound of the flute and the cithera has ceased; there is no animated talk of the last winners at the Isthmian or the Olympian games; the clatter of the loom and the domestic hum of the spinning-wheel are no longer heard; the naked feet of the slaves and the women fall carefully and silently upon the uncarpeted floors, and an unwonted stillness reigns throughout the numerous apartments of the dwelling. There is no savory steam of roasting wild-boar from the kitchen, and the fragrant Thracian wine stands untasted on the table, with a few plain barley-cakes and a little salt fish.

"Silenus lies in his sleeping chamber, in the quiet interior part of the house, adjoining the apartments of the women, farthest from the vestibule, and near to the garden. By the bed of the sick man, there is a small tripod stand, with a circular top, and upon it there is a statuette of Hercules, a bowl of warm barley-water, and a cup of oxymel.

"Leaning her head on the foot of the bed and sobbing, sits, on a low stool, a young Greek woman, beautiful in her features, and graceful in the flowing outlines of her person, as the Thessalian maidens of Homer. There is a picturesque combination of barbarian rudeness and Grecian elegance in her appearance, not an unfitting type and expression of the age and state of society in the midst of which she lived. Her feet and ankles are bare; she wears only a single garment—the long Ionic chiton of linen—with large sleeves reaching only a little below the shoulders, leaving uncovered, in their snowy whiteness, arms that might have rivalled those of the jealous queen of Olympus. A girdle fastens the robe loosely round a waist, like that of the Medician Venus, innocent of the deformities of buckram and whalebone. The light auburn hair is simply parted and carried back from the forehead, gathered in a knot on the crown of the head, fastened with a golden grass-hopper, and held by a coif of golden net-work.

"At the head of the bed, watching steadfastly and earnestly the appearance of the patient, is seated his physician, the already celebrated son of Heraclides and Phenaretes, Hippocrates of Cos. He has just entered the apartment, to make his morning visit. His sandals have been taken off, and his feet washed by a slave in the vestibule. He wears over his linen tunic a large flowing mantle of light fine woolen, suited to the season, not unlike the later toga of the Romans, fastened at the neck with a cameo of Æsculapius, and falling in graceful folds nearly to his feet. His hair is long, and both this and his beard are kept and arranged with scrupulous neatness and care. He is thirty years old, in the very prime and beauty of early manhood. His features, through these misty shadows of many centuries, we cannot clearly distinguish, but we see that his face is dignified, thoughtful, and serene; and his whole aspect, manner, and expression, are those of high, antique breeding, of refined culture, and of rather studied and elaborate elegance.

"His examination of his patient was long, anxious, and careful. He saw at once that the gravity and danger of the disease had increased since his last visit. He inquired very minutely into the manner in which the night had been passed; and was told by the watchers that the patient had had no sleep, that he had talked constantly, had sung and laughed, and had been agitated and restless. He found the hypochondria tumefied, but without much hardness. The stools had been blackish and watery, and the urine turbid and dark-colored. He noticed the temperature and feel of the skin, and he studied for a long time and with great solicitude the general manner and appearance, the decubitus, the breathing, the motions, and especially the physiognomy of the patient. The only circumstance in the examination that would have particularly attracted the attention of a modern witness of the scene, would have been his omission to feel the pulse. With this exception, no examination of the rational symptoms of disease could have been more thorough and methodical.

"Having satisfied himself as to the state of his patient, he retired to an adjoining room, followed by some of the attendants, to give directions in regard to the few simple remedies that he intended to use. The patient had already been bled, and had had a purgative of black hellebore. Hippocrates directed that, instead of the strained decoction of barley, which had been the patient's drink, he should now have honey and water—the favorite hydromel—that the bed should be made softer—the windows of the room still farther darkened, and that a warm flax-seed poultice, softened with olive-oil, should be applied to the abdomen.

"With a sad but decided expression of his fears as to the issue of the case, and a few kindly and pious words to the weeping wife, about the dignity, the solace, and the duty, in all our trials, of submission to the will of the gods, he gathered his mantle gracefully about him, had his sandals refitted by the slave who waited in the vestibule, and proceeded on his daily round of visits among the houses of the city."

The young physician of Thasos is next seen by the bedside of the dying Pericles.

"Philosophy, letters, and art, breathe in the quiet atmosphere of the room; and the taste of Aspasia sheds an Asiatic grace over its furnishing and its decorations. In one corner stands a statue of Minerva, from the chisel of Phidias; and the walls are covered with pictures, fresh from the pencils of Panænus and Polygnotus, illustrating the legendary and historic glories of Greece. There might have been seen Theseus, bearing off from the field of victory, on the banks of the Thermodon, the masculine and magnificent queen of the Amazons—half willing, perhaps, to be the captive of such a victor; Jason, in his good ship Argo, with his fifty selectest heroes, convoyed by the queen of love, the awful Here, and Apollo, winds his various and adventurous voyage, crowded with poetic imagery and romantic incident, and brings back the golden fleece from Colchis;—Helen, at her loom, is weaving into her 'golden web' the story of the Trojan wars;—the chaste Penelope, by the light of her midnight lamp, undoes the delusive labors of the day;—Ulysses, returned from his long wanderings, surveys once more, with boyish pride and delight, the dear old bow, which no arm but his could bend. . . . . . . . . . . . . .

"Not often in the world's history has there met together a more august and illustrious company. There are a few of those whom we are able to recognise amongst them. Resting his head on the shoulder of Socrates, and sobbing aloud in unrestrained and passionate sorrow, leans the wild and reckless Alcibiades—just in the first bloom of that resplendent personal beauty which made him seem to the eyes, even of the Greeks, more like the radiant apparition of a young Apollo, than any form of mere earthly mould—subdued, for the first time in his life, and probably for the last—by the spectacle before him, of his dying relative and guardian—to reverence, tenderness, and truth. Sophocles, his old companion in arms, is there; and near him, in his coarse mantle, and with unsandaled feet, may have stood a grandson of Aristides, still poor with the honorable poverty of his great ancestor.

"Conspicuous amidst this group of generals, admirals, statesmen, orators, artists, poets, and philosophers,—in rank and fortune, in social position, in reputation, in learning, culture, and refinement, their equal and associate, sits the young physician of Cos. Already had his rising fame reached Athens, and when the city, overcrowded with the inhabitants of Attica, driven from their homes by the armies of Sparta, was smitten with the pestilence, he was summoned from his island home in the Ægean, to stay, if he could, the march of the destroying angel, and to succor with his skill those who had fallen under the shadow of its wings."

The titles of the works now regarded as from the pen of Hippocrates are:—

" 1. Ancient Medicine.
" 2. Prognosis.
" 3. Aphorisms.
" 4. Epidemics, 1st and 3d Books.
" 5. Regimen in Acute Diseases
" 6. Airs, Waters, and Places.
" 7. Articulations.
" 8. Fractures.

" 9. Instruments of Reduction.
" 10. Wounds of the Head.
" 11. The Oath.
" 12. The Law."

We have short notices of each of the works, with extracts from some of them, showing the condition of medicine in this earliest period of its history. We must close with one extract, which we commend to those who are constantly croaking about the degeneracy of our times, and the ignorance and inefficiency of the profession :—

" The science of medicine is, historically, twenty-two centuries old. Since its origin in Greece, four hundred years before the Christian era, it has never ceased to be cultivated, wherever any considerable degree of civilization has been reached. During all this long period, the science of medicine, like its kindred sciences of observation, has obeyed its own inherent and vital law of development. Subject always to its various and complicated relations ; sometimes seduced or driven from its true path ; sometimes obstructed or hindered in its march ; sometimes dragged backward, it has still steadily struggled onward, obedient to the living principle of growth and progress within it. It has experienced the same vicissitudes ; it has encountered the same obstacles and hindrances ; it has achieved the same triumphs, as its sister sciences, —as astronomy, meteorology, geology, and chemistry ; and the like common destiny of glory and beneficence awaits it and them.

" It is natural enough, when we look at the popular medical delusions of our day, and the skepticism as to the claims of medical science and art, which has seized upon the minds even of sensible and cultivated men,—that we should have some misgivings as to the permanency and stability of this science and art. But the great organic laws of nature are not to be suspended, nor reversed, nor turned aside. The lessons of twenty-two centuries are not to be forgotten, nor made to contradict themselves, for the first time, to-day. The science is constituted by the results of the toilsome and conscientious study of nature during these long centuries, recorded, systematized, and arranged ; and as long as nature remains what it was two thousand years ago, and what it is to-day, these results will remain. The art is the practical application of the science ; it has been the chief minister to sick and suffering humanity, in all ages, and amongst all civilized people ; it is so to-day,—it will be so to-morrow. What has been will continue to be. The laws, ordained at the beginning, will still rule over us. The sun that shone upon Athens, and upon Pericles, shines still upon us ; and it will continue to shine upon all who are to come after us. Spots may sometimes pass over its surface, but they do pass, and they neither dim nor darken the radiance of its disc. Clouds and mists may intercept, for a season, its beams; but it is only for a season; and the Hand that hung it in the heavens will still maintain it there, to bless the future, as it has blessed the past, with its kindly and beneficent light." J.

